# Motivations, acceptability and ethical considerations for interventional HIV cure research at the end of life: perspectives from long-term survivors of HIV in the United States

**DOI:** 10.1186/s12910-025-01253-x

**Published:** 2025-08-23

**Authors:** Ali Ahmed, Jeff Taylor, Whitney Tran, Simran Swaitch, Samuel O. Ndukwe, Rachel Lau, Kris H. Oliveira, Stephanie Solso, Cheryl Dullano, Andy Kaytes, Davey Smith, Robert Deiss, Sara Gianella, Karine Dubé

**Affiliations:** 1 Division of Infectious Diseases and Global Public Health, School of Medicine, University of California San Diego (UCSD), 9500 Gilman Drive, La Jolla, CA 92093 USA; 2 HIV + Aging Research Project – Palm Springs (HARP-PS), Palm Springs, CA USA; 3 Reversing Immune Dysfunction for HIV-1 (RID-HIV) Eradication Martin Delaney Collaboratory Community Engagement Coordination and Community Advisory Board, San Diego, CA USA; 4 Departamento de Infectologia e Medicina Tropical, Faculdade de Medicina da Universidade de São Paulo (FMUSP), São Paulo, Brasil; 5 UCSD AntiViral Research Center Community Advisory Board (CAB), San Diego, CA USA

**Keywords:** HIV cure research, End of life, Interventions, Analytical treatment interruptions, People with HIV, Long-term survivors, Ethics

## Abstract

**Introduction:**

Despite progress in antiretroviral therapy (ART), an effective cure for HIV remains out of reach. End-of-life (EOL) research studies involving individuals with a prognosis of six months or less offers an opportunity to advance cure science but has so far been limited to observational designs focused on HIV reservoirs. As interventional approaches at the EOL are now being considered, it is essential to assess their acceptability before moving forward. Understanding how long-term survivors (LTS) of HIV perceive these potential interventions, along with their motivations and ethical considerations, is critical to guiding the design of future EOL-HIV interventional research.

**Methods:**

We conducted in-depth qualitative interviews with 16 LTS of HIV from across regions in United States to examine their views on hypothetical interventional HIV cure research at the EOL. To ensure representation, we recruited participants through community-based organizations and HIV cure collaboratories using purposive-sampling. We conducted interviews via secure teleconferencing, transcribed the recordings, and used inductive thematic analysis to identify key themes related to motivations, acceptability, and ethical considerations surrounding interventional EOL HIV cure research.

**Results:**

Participants viewed hypothetical interventional HIV cure research at the EOL as a way to contribute to science, despite expecting no personal benefit. They prioritized autonomy and informed-choice in ethical participation. Many supported latency-reversing agents but raised safety concerns; in contrast, they viewed block-and-lock strategies as promising and less risky. Participants generally welcomed immune-based approaches, though some questioned their suitability for older adults near the EOL. LTS found cell and gene-based interventions innovative but expressed caution about safety and feasibility. While they valued the scientific potential of combination strategies, they noted their complexity and burden. Opinions on analytical treatment interruptions were mixed and depended on ethical safeguards, including medical oversight and reversibility. Willingness to participate in hypothetical HIV cure research at the EOL reflected individual health status, perceived burden, and personal values.

**Conclusions:**

LTS are willing to engage in interventional EOL HIV cure research but emphasize the importance of ethical safeguards and participant autonomy. As HIV cure research progresses, integrating LTS perspectives is critical to designing feasible, ethical, and scientifically impactful interventional studies at EOL.

**Supplementary Information:**

The online version contains supplementary material available at 10.1186/s12910-025-01253-x.

## Introduction

Safe and effective antiretroviral therapy (ART) has transformed HIV from a fatal disease into a manageable chronic condition; however, a cure remains a scientific goal [1]. For people with HIV (PWH), particularly long-term survivors (LTS), who were diagnosed before the widespread availability of effective ART in the mid-1990s, at a time when an HIV diagnosis was considered a death sentence, the pursuit of an HIV cure represents not only a scientific imperative but also a profoundly personal journey [2]. Having lived through the epidemic’s early years, LTS endured the loss of loved ones and often faced their own mortality in the absence of effective treatment [3, 4]. Many LTS either participated in early clinical trials of experimental therapies like azidothymidine monotherapy or independently sought out unproven treatments such as dinitrochlorobenzene or ribavirin in efforts to stay alive [5–7]. Today, they seek to give back by contributing their lived experiences to HIV cure research [3]. 

Research into HIV cure strategies has intensified in recent years [1, 8]. However, many of these interventions remain in experimental stages, and their risks and feasibility are still being evaluated [9]. End-of-life (EOL) research typically involves people in the final months of life with a prognosis of 6 months or less, who voluntarily participate in studies to help advance science [10, 11]. In other disease areas, such as cancer and neurodegenrative disorders, individuals nearing the EOL have participated in trials to test experimental therapies, study drug delivery, or evaluate treatment safety [12–14]. EOL research represents a promising opportunity to advance HIV cure science, yet it remains limited to observational studies focused on understanding viral reservoirs, areas in the body where HIV persists despite ART treatment [15, 16]. However, there is growing interest in exploring interventional approaches during this stage of life, especially among LTS, whose lived experience and immune profiles offer unique scientific insights. These efforts have led to important scientific discoveries and demonstrate that meaningful, ethical research is possible even in the final stage of life [17]. While interventional EOL HIV cure research is still hypothetical, it may soon become a reality. Assessing its ethical and practical acceptability is essential before moving forward with interventional trial implementation. The inclusion of LTS in EOL cure research is particularly insightful given their distinct medical and psychosocial histories. Many LTS have lived with HIV for decades, experiencing prolonged exposure to ART, immune dysregulation, and cumulative social stigma [3, 18]. Their aging process is often accelerated due to chronic inflammation and comorbidities, making their participation in cure research even more complex [19]. Older PWH are often excluded from clinical trials due to concerns about frailty, life expectancy, and lack of direct clinical benefit, limiting the generalizability of cure research [2, 20, 21]. Understanding LTS perspectives on hypothetical interventional HIV cure studies at the EOL is critical for shaping ethical guidelines and ensuring research inclusivity.

EOL research presents a unique ethical and scientific opportunity, particularly for studying the persistence of HIV reservoirs in the human body [11, 15, 22–25]. Unlike traditional HIV cure studies with otherwise healthy volunteers [26], which require long-term monitoring and careful risk-benefit considerations for participants, EOL research allows for a different ethical framework, one that prioritizes the participant’s autonomy while maximizing scientific insight [15, 25]. Previous observational research, such as the Last Gift study, at the University of California San Diego (UCSD), has shown that altruistic individuals nearing the EOL are willing to contribute to HIV cure research, including tissue donation or rapid research autopsy-based studies that examine viral reservoirs in various tissue compartments across the entire body [11, 15, 22–25]. Participants in the Last Gift study are not dying of AIDS. Rather, they are PWH with another terminal illness such as cancer, neurodegenerative, or cardiovascular disease and have a prognosis of six months or less [10, 11, 15, 22–25]. In 2019, the Last Gift study protocol was amended to include PWH with a chronic condition and multiple co-morbidities associated with a five-year mortality of greater than 50% [27] A key feature of the Last Gift protocol is the rapid research autopsy, conducted within six hours of death, which enables high-quality virologic and immunologic analyses [28, 29]. Nevertheless, there remains a significant gap in understanding how LTS themselves perceive interventional EOL HIV cure research, where participants might not only donate their tissues for scientific analysis but also engage in testing experimental interventions that could inform future cure strategies [10]. 

Another major gap in the literature is the lack of data on LTS perspectives regarding specific HIV cure strategies in the context of EOL research. Different HIV cure research approaches vary in both risk and feasibility. Latency-reversing agents (LRAs) aim to awaken dormant HIV, but may pose immunological and clinical risks for older PWH [9]. Meanwhile, block-and-lock strategies, which attempt to permanently silence the virus rather than eliminate it [30, 31], may offer a more stable but less definitive alternative to a cure. Immune-based therapies [32], such as broadly neutralizing antibodies (bNAbs) and therapeutic vaccines, hold promise, though their efficacy in immune systems of aging people is uncertain. Cell and gene therapies [33], including Clustered Regularly Interspaced Short Palindromic Repeats (CRISPR) [34], raise additional ethical and long-term safety concerns. Understanding how LTS evaluate these options in the EOL context is essential for guiding future study designs and ensuring that research aligns with participant and community priorities. While interventional EOL HIV cure studies may not be designed to fully assess efficacy given participants’ limited lifespan and the small scale of such trials, they offer a unique and powerful opportunity to study how interventions behave in the human body. Specifically, these studies can provide critical insights into how experimental agents distribute across tissues and deep anatomical compartments where HIV persists, such as the brain, gut, lymph nodes, and genital tract. This level of access is rarely possible in traditional studies with living participants. Interventional EOL research can also help elucidate mechanisms of action, immune responses, and potential off-target effects in real-time. Such data are essential for optimizing future cure strategies and de-risking interventions before broader testing in otherwise healthy individuals.

A key ethical concern in interventional EOL research is balancing the risk tolerance of potential participants with the need to obtain informed consent [35]. Unlike younger participants who might weigh trial participation against long-term survival prospects, EOL participants often frame their decision-making around legacy, contribution to science, and the potential to help future generations [36, 37]. Prior research on altruism in HIV studies has shown that many PWH are willing to take personal risks if their participation advances science [38, 39]. However, interventional research at the EOL raises additional ethical and logistical questions: how do LTS perceive the risks of experimental interventions near the EOL? Would they be willing to interrupt ART to support the search for an HIV cure? What safeguards do they believe are necessary to ensure their autonomy and well-being? Answering these questions is critical for designing ethical and meaningful HIV cure studies at the EOL.

Questions surrounding the acceptability of analytical treatment interruptions (ATIs) at the EOL remains underexplored. Many cure studies require ATIs to assess whether an intervention is able to suppress HIV without ART [40, 41]. However, discontinuing ART can lead to viral rebound and exacerbate health complications [42]. While some participants at the EOL may view ART discontinuation as an acceptable risk, others may value the certainty of ART adherence in their final months [43]. Building on the success of observational EOL studies [11, 15, 23, 24], there is growing interest in interventional approaches where PWH at the EOL will undergo procedures solely to advance cure science, without expectation of personal clinical benefit to their HIV or terminal illness. Moreover, this group presents a unique opportunity, as ART interruption would have fewer clinical consequences. Further, participation in this context is driven by altruism. Understanding LTS’ views on possible interventions, ART interruption, and research participation is essential to assess feasibility and guide ethical safeguards. Moreover, our team at UCSD is proactively conducting formative ethics research, and engaging diverse perspectives from PWH across the United States (U.S.) is critical to ensure future studies are responsive, inclusive, and ethically grounded [10, 11, 15, 22–25]. This study employed qualitative interviews with diverse LTS of HIV to explore their motivations, concerns, and perspectives on hypothetitcal interventional HIV cure research at the EOL. Our findings provide critical insights into how LTS navigate the intersection of personal health, scientific contribution, and decision-making around EOL interventions in the context of innovative HIV cure research.

## Methods

### Study design

We employed a qualitative approach to observe the nuanced and deeply personal perspectives of LTS of HIV about hypothetical interventional HIV cure research at the EOL [25, 44]. This method provided rich, contextual insights and enabled an in-depth exploration of their experiences. Given the limited research on this topic, qualitative design was essential to uncover diverse perspectives, ethical considerations, and motivations, addressing a critical gap in the literature [11]. 

### Study setting and participants

This study (Clinical Trial Number: Not Applicable) was part of a larger U.S.-based project examining LTS perspectives on HIV cure research [45]. For the hypothetical EOL interventional research component, we conducted in-depth interviews (IDIs) with 16 PWH willing to discuss the sensitive topic of HIV cure research at EOL, ensuring diverse representation of LTS across U.S. regions. Recruitment was facilitated through personal contacts with community-based organizations (CBOs) and academic partnerships, including AIDS Action Baltimore, the HIV + Aging Research Project — Palm Springs (HARP-PS), and the Reunion Project. We also partnered with community engagement partners and community advisory board (CAB) members affiliated with three Martin Delaney Collaboratories for HIV Cure Research: (1) Delaney AIDS Research Enterprise (DARE), (2) Reversing Immune Dysfunction for HIV-1 Eradication (RID-HIV), and (3) CRISPR for Cure who assisted with sharing recruitment materials through their networks. While some participants had prior exposure to HIV cure research through advocacy or community roles, many were not directly involved in scientific research. To help reduce potential bias and broaden perspectives we also partnered with other CBOs serving aging PWH, such as the NMAC (formerly known as National Minority AIDS Council), The Well Project, and the San Francisco AIDS Foundation (SFAF). To enhance diversity, we distributed an institutional review board (IRB)-approved flyer and asked HIV and aging specialists to refer LTS from diverse backgrounds. This purposive, non-probabilistic sampling approach was designed to capture a broad range of perspectives. Participants understood that joining this study did not imply future participation in EOL clinical research. By the 16th interview, we reached data saturation, as no new themes emerged, confirming the depth and comprehensiveness of the perspectives gathered [46, 47]. 

### Interview guide

We designed a semi-structured interview guide (Table 1) in collaboration with our Last Gift community collaborators to explore LTS’ perspectives on hypothetical interventional HIV cure research at EOL. The IDI guide explored whether PWH approaching EOL should have the opportunity to enroll in these innovative studies, given their altruistic nature. It also explored views on specific interventions, including LRAs, block-and-lock strategies, immune-based approaches, cell and gene therapies, and combination approaches. Additionally, the guide addressed the acceptability of ATIs at EOL, assessing potential concerns, and necessary safeguards.

**Table 1:** IRB-approved interview guide: perspectives of diverse long-term survivors of HIV on interventional HIV cure-related research at the end of life (2023–2024)

**Introduction**
First, thank you so much for your time in participating in this interview about HIV cure-related research at the end of life, which we are now recording.
**Acceptability of Testing Interventions in People with HIV at the End of Life**
To date, end-of-life HIV cure-related research has been limited to observational assessments – for example studying what happens to HIV reservoirs deep inside the body when ART is maintained or interrupted by the study participant. This type of research is providing scientists with priceless insights into the HIV reservoir that cannot be gained in any other way. This is the reason for the study name. Through their last gift of permitting study of their bodies after death, participants make a unique contribution towards an HIV cure for others in the future.
Because of the success of these *observational* end-of-life HIV cure studies, there is now growing interest in the possibility of testing HIV cure *interventions*. PWH would undergo study interventions, or procedures, solely to advance HIV cure-related research, not in the hope of staying alive or prolonging life. PWH enrolling in this type of research would do so entirely for altruistic reasons, to help others and with no expectation of medical benefits to themselves for their HIV or other illness.
• In general, do you think people with HIV who are approaching the end of life should be offered the opportunity to participate in interventional studies? Why or why not?
• Next, I will describe briefly several types of such interventional studies. Please tell me your views about offering the opportunity to participate in each type.
o Latency-reversing agents (compounds that would reawaken HIV that has become dormant inside the cells so that the virus can be targeted for clearance by an intervention or therapy)
o Immune-based strategies (approaches that are aimed to strengthen the immune system)
o Cell and gene approaches (approaches that are aimed to make cells resistant to HIV infection and may involve gene changes that would not be passed to your children)
o Block and lock strategies (approaches that block reactivation of HIV in dormant cells without eliminating them, then locks them in that sleeping state)
o Combination approaches
• Do you think participants in these types of studies should have the option, as some PWH desire, to interrupt HIV treatment as they approach the end of life?
o Why or why not?
o What types of safeguards should be put into place for underdoing HIV treatment interruptions at the end of life?
**Closing**
• Is there anything else you would like us to know about HIV cure-related research at the end of life?

### Data collection

After participants confirmed their willingness to participate in IDIs with the study’s principal investigator (K.D.), the research team provided them with an IRB-approved informed consent form, a demographic survey, and an IDI guide. Two members of the research team (K.D. and J.T.) conducted and documented the interviews using a Health Insurance Portability and Accountability Act (HIPAA)-compliant teleconferencing platform (Zoom). All interviews took place in English via audio and/or video teleconference, depending on participants’ preferences, ensuring privacy and security. Upon completing their interview, participants received a $50 Visa gift card, either in a virtual format or as a physical card delivered by mail based on their preference.

### Data analysis

All interview audio recordings were stored on a restricted-access drive, available only to the research team. These recordings were later transcribed using an encrypted transcription service. To ensure accuracy and completeness, research assistants (W.T., S.S.) carefully reviewed the audio recordings and transcripts, removing any identifiable information. Any inconsistencies were addressed by cross-referencing the transcripts with the original recordings. Participants did not have access to the transcripts or study findings.

For qualitative data analysis, we conducted a conventional thematic analysis using an inductive approach [46]. De-identified responses were compiled into a master document, systematically arranged by interview question, and prepared for manual coding. Given the exploratory nature of this study, no predefined coding framework was applied. Instead, the primary coder (A.A.) developed the initial codebook and identified key excerpts. A secondary review was conducted by the principal investigator (K.D.), who refined the coding structure and organized excerpts into a tabular format. Both researchers used inductive methodologies to identify emerging themes and subthemes. Discrepancies in coding were addressed through collaborative discussions held bi-weekly. After reviewing the data, the research team concluded that thematic saturation had been achieved [48]. 

Representative quotes reflecting key themes are provided in the results section, with further examples included in Supplementary Table S1.

### Ethics statement

This study was conducted in accordance with all applicable ethical guidelines and regulatory standards, including the U.S. Code of Federal Regulations and the Declaration of Helsinki. The UCSD IRB granted approval for the study (IRB #808882). Prior to participating in interviews, individuals were provided with an informed consent document. Their verbal consent was captured during the interview process, in compliance with IRB approval. To ensure participant confidentiality, all study-related materials, including interview transcripts, were de-identified, and the original audio recordings were securely deleted after the transcription quality was verified.

## Results

This study included 16 LTS of HIV (mean age: 68.4 years, range: 60–82), including nine (56.25%) cisgender men and 7 (43.75%) cisgender women. Racial identities included White/Caucasian (56.25%), Black/African (25%), Asian (including Indian) (12.5%), and mixed race (6.25%). More than half (56.25%) held a college degree. Participants lived across the West (50%), South (31.25%), Northeast (12.5%), and Midwest (6.25%) regions of the U.S. They had a mean age of 27.1 years (SD: 10.5) in HIV-related work or advocacy, and 7.3 years (SD: 9.8) in HIV cure research. LTS year of HIV diagnosis ranged from 1985 to 2007, with 13 participants (81.25%) diagnosed before the introduction of effective ART in 1996. (Table 2) Although the study aimed to recruit individuals diagnosed during the pre-ART era, a few later-diagnosed participants, particularly women, were included to enhance gender diversity and reflect the broader spectrum of long-term survivorship.

**Table 2:** Demographic Characteristics – long-term survivors of Hiv on cure research at the EOL (United states, 2023–2024)

Characteristics	*N* (%)
Age in years, mean (min-max)	68.4 (60–82)
Sex assigned at birth	
Male	9 (56.25)
Female	7 (43.75)
Gender	
Cisgender Man	9 (56.25)
Cisgender Woman	7 (43.75)
Education	
Less than high school	1 (6.25)
High school diploma or GED	1 (6.25)
Some college, but no diploma	4 (25)
College graduate	9 (56.25)
Master’s degree or equivalent	1 (6.25)
Race	
White/Caucasian	9 (56.25)
Black African/American	4 (25)
Asian	2 (12.50)
Mixed	1 (6.25)
U.S. Region	
Northeast	2 (12.5)
Midwest	1 (6.25)
South	5 (31.25)
West	8 (50)
Years worked in HIV related advocacy in general, mean (std. dev.)	27.13 (10.46)
Years worked in HIV Cure related advocacy, mean (std. dev.)	7.34 (9.78)

The first section of the results explores motivations for participating in hypothetical interventional HIV cure research at the EOL, emphasizing altruism and the perceived scientific value. This is followed by perspectives on autonomy and informed choice, highlighting participants’ views on making decisions about research participation. The next sections examine participants’ perceptions of various cure strategies, including LRAs, block-and-lock strategies, immune-based approaches, cell and gene therapies, and combination approaches. Each strategy is discussed in terms of interest, concerns, and perceived feasibility at the EOL. Subsequently, the results show diverse opinions on ATIs at the EOL, including both support and concerns, along with possible safeguards.

## Motivations and autonomy in research participation in interventional HIV cure research at the EOL

### Altruism and contribution to science

Most participants expressed a desire to contribute to HIV cure research, emphasizing their willingness to participate even if they would not personally benefit. LTS viewed their involvement as a means to support efforts toward an HIV cure, recognizing the progress made in extending their lives and hoping to help future generations.

*A lot of us hoped, especially long-term survivors… that we would live long… basically normal life and a lot of us have, and so [we would] like to be a part of something that could end up being a cure. *– Cisgender Man, White.

*If we don’t do the testing and research… how would we ever know if it might’ve helped someone in their final days? For me, the most important part is doing all these things you mentioned, so the next person might benefit. So yes, I would say I support it.* – Cisgender Female, Black.

Some participants expressed a willingness to donate their tissues for interventional research at the EOL, with the understanding it would not cure their HIV or the terminal illness they faced. Others emphasized the broader significance of advancing HIV cure science, believing that many PWH in their position would be eager to contribute to efforts towards achieving durable virologic control without need of ART.

### Scientific value and willingness to participate

LTS recognized the unique scientific value of conducting interventional studies at the EOL, believing that such research could lead to groundbreaking discoveries. They viewed this as an opportunity to contribute to HIV cure efforts in ways that traditional observational studies could not achieve. Participants opposed the exclusion of PWH at the EOL from interventional studies, arguing that it can reinforce HIV-related stigma and diminish their worth. They emphasized the importance of informed consent, believing that as long as PWH understand the risks, they should not be disqualified from participation. Some expressed openness to trying experimental interventions, if the risks were minimal.

*I actually think it’s kind of stigmatizing to not allow me to participate in a study because basically, what you’re saying is, I’m at the end of my life, and I have no value anymore.* – Cisgender Man, White.

Participants emphasized that PWH at the EOL should have the right to contribute to HIV cure research if they wish.

*Unless there is overriding evidence that this is going to be harmful to me… there’s none of these that I think should be disallowed from somebody having the opportunity to try it.* – Cisgender Man, White.

### Autonomy and informed choice

Participants emphasized the importance of personal autonomy and the ability to make informed decisions about participation in interventional studies at the EOL. They believed PWH should have the right to decide whether to engage in such research, particularly when facing EOL decisions. Many participants stressed that decisions about research involvement should be left to the individual, provided they are mentally competent and fully informed.

*It is my life; it is my choice. As long as I am mentally competent to make a decision, this is my choice, I mean, to knowingly go into a trial that may not help me.* – Cisgender Man, White.

Participants highlighted the need for clear communication about the purpose and potential risks of EOL studies, emphasizing that discussions should be led by knowledgeable professionals. Beyond clinical monitoring, participants stressed the importance of emotional support in decision-making. They wanted clear communication to ensure that decisions were made by the individual. Some expressed openness to more invasive interventions if the risks were acceptable.

*As long as I fully understand what I’m signing up for, there’s none of them that I think should be disallowed.* – Cisgender Man, White.

By emphasizing autonomy, informed decision-making, and the right to weigh risks, participants reinforced the belief that individuals should have control over their participation in interventional HIV cure research at the EOL.

### Acceptability of testing specific HIV cure research interventions at the EOL

#### Perceptions on testing LRAs at the EOL

While this section focuses on perspectives regarding the acceptability of testing LRAs at the EOL, several participants framed their views more broadly in relation to aging with HIV. For many LTS, aging and EOL were deeply interconnected experiences shaped by decades of illness, uncertainty, and treatment fatigue. As such, their concerns often reflected both the risks of LRAs for older people and their potential implications near the EOL.

Participants who supported LRA studies at the EOL emphasized their potential to advance HIV cure research. They viewed these studies as crucial for understanding HIV persistence and elimination, advocating for continued efforts to awaken and clear latent reservoirs rather than abandoning the approach.

*I would be interested in it [LRA] because it would tell you something.* – Cisgender Female, Mixed Race.

Skepticism about LRA studies emerged among participants who feared that reactivating latent HIV could trigger uncontrolled viral replication, threatening health and treatment stability. Participants who had maintained undetectable viral loads for years were particularly hesitant, citing concerns about viral adaptability and resistance.

*To me, sounds like you’re wakening the beast.* – Cisgender Man, White.

*Why would I want to take a chance of my virus waking up and killing me? I’ve been undetectable since ‘97… If somebody wakes it up, and from what I know about the virus, it’s very smart… No, I would not do it.* – Cisgender Female, Asian.

Others voiced broader concerns about reactivating latent pathogens, heightened vulnerability with aging, and increased risk of opportunistic infections. Given these uncertainties, several LTS suggested LRA studies should first be tested in younger, immunologically resilient populations before involving older PWH. Fears of a shortened lifespan and irreversible health effects deterred participation, suggesting potential recruitment challenges, particularly among older LTS.

*The issue with latency-reversing agents is if they’re going to reverse HIV latency, which other pathogens that are latent might also have their latency reversed? They should perhaps only use latency-reversing agents if there’s already clinical data in younger participants.* –, Cisgender Man, White.

Despite these concerns, some participants remained open to LRA studies, recognizing their scientific value. They were curious about how these interventions worked and viewed them as opportunities to deepen understanding of HIV persistence and latency reversal. Even while acknowledging the risks, they expressed interest in contributing to research that could ultimately benefit others.

#### *Perceptions about block and lock approaches at the EOL*

Many participants viewed block and lock as an innovative and potentially less risky HIV cure approach. Unlike other strategies that attempt to eliminate the virus, block and lock aim to suppress HIV indefinitely by keeping it in a dormant state. This concept resonated with several participants, who viewed it as an attractive and feasible method as it attempts to replicate exceptional elite control of HIV. Some expressed curiosity about how block and lock would work at a biological level and showed interest in its potential application.

*I think that’d [block and lock] be a good approach. That’s interesting. I wonder how they would do that. Because then you’re getting into molecular biology. So and that’s really advanced, I would go for that one. Yeah. – Cisgender Man, White *

*Cisgender Man, White*.*Absolutely it’s [block and lock] less risky, perhaps… I think it’s certainly acceptable. I think it’s certainly something that people would do. I would.* – Cisgender Male, White.

Participants questioned the feasibility and long-term effectiveness of block and lock strategies, particularly whether the “lock” would remain effective and if older PWH at the EOL would be included in research. Some found the concept unsettling, fearing the virus could eventually break free or cause unforeseen consequences. Despite skepticism, participants acknowledged potential benefits for some and supported continued research on block and lock approaches.

*I mean, the thing with block and lock is how long does the lock remain effective? And I imagined researchers would want people who were relatively young and healthy… [T]hey [these studies] might not be particularly attractive to people who don’t have very long to live anyway.* – Cisgender Man, White.

#### *Perceptions about immune-based strategies at the EOL*

Many participants strongly supported immune-based HIV cure strategies, reflecting their deep belief in the body’s natural ability to fight disease. They viewed interventions such as bNAbs and vaccine-based approaches as both promising and acceptable at the EOL, particularly given the absence of safety concerns. These strategies were seen as aligning with a broader interest in restoring or enhancing immune function, something participants noted was important for healthy aging and overall well-being. Some suggested that immune-based research could have broader relevance beyond HIV, including applications in cancer and other chronic illnesses.

*It sounds particularly appealing to me, because I think that our bodies are pretty amazing. And I’ve always been all about, like, having a good healthy immune system… Maybe some of that work could translate into cancer research too.* – Cisgender Female, White.

*No problems with that at all. I see no particular problem. And using broadly neutralizing antibodies, and vaccine, vaccine therapies, those sorts of human-based services, definitely, they should be good.* – Cisgender Man, White.

However, others expressed more cautious or mixed views. While intrigued by the concept, some questioned the relevance and safety of immune-based strategies for older adults, particularly given concerns about immune senescence. They called into question whether these interventions were better suited for younger populations and raised concern about the unintended effects of boosting the immune system, including the risk of inadvertently accelerating viral replication.

*Well, I remember when I was newly diagnosed, they were saying that if you do something to increase your immune system, the virus also gets that system with it. So, does that mean the virus is going to be assessed to also to fight off? The T cell? I don’t know. Unless they can just do the T cell and not break that HIV. The virus in itself, but I don’t know how we’re done.* – Cisgender Female, Asian.

Despite some uncertainties, participants generally viewed immune-based interventions with cautious optimism. Participants appreciated the immune-based interventions, especially if they could harness the body’s natural defenses to clear HIV without causing harm.

#### *Perceptions about cell and gene approaches at the EOL*

When asked about cell and gene-based interventions, most participants responded positively, expressing interest in contributing to research that could shape the future of HIV cure science. Although participants often used the terms interchangeably, their opinion involved a range of biomedical strategies, including cell therapies (modification of immune cells), gene therapies (genetic alterations to confer viral resistance), and gene editing technologies such as CRISPR. While these approaches differ in their scientific definitions with cell therapies involving cellular manipulation, whereas gene therapies directly target the genome, participants broadly regarded them as interconnected and promising avenues for advancing HIV cure research. Participants viewed the EOL context as an appropriate setting to test interventions that may be considered too risky for younger or healthier individuals. Some noted that long-term side effects were less of a concern at this stage of life, making older PWH uniquely positioned to support early-phase testing.

*These would be ones that I think should be especially considered for end-of-life participants. Especially if they aren’t regarded as perhaps a little too risky to do in younger, healthier people.* – Cisgender Man, White.

*Yeah. I think these could be particularly valuable in older people, because some of the concerns are, you know, the off-target reactions, but if you’re at the end of life anyway, it’s, you know, the consequence of, you know, off-target effects might not be as great. So, yeah, I mean, the promise of, you know, the sort of gene editing approaches could have really dramatically effective responses.* – Cisgender Man, White.

Participants voiced concerns about the safety and uncertainty of gene editing, with some unwilling to participate at the EOL due to unproven results and potential risks. Others were skeptical about its long-term effects and viewed it as a gamble. Ethical worries also emerged, with comparisons to designer genetics. Some LTS were willing to accept higher risks in HIV cure research, including gene therapy, emphasizing the need for testing and research to advance future clinical benefits for future generations of PWH.

Some participants questioned whether gene therapy would take effect quickly enough to produce a meaningful difference for PWH at the EOL. Given the advanced stage of illness, they expressed concerns that any potential therapeutic benefits might not manifest before death. This raised doubts for some about the personal relevance of participation.

*I don’t, I mean, because you know, how long will it take for these [interventions] to show a difference in the person and in stage, before they die, before it takes effect. Does it make sense? You have to determine how long it’s going to take for to see show any progress.* – Cisgender Female, Black.

### Perceptions about combination approaches at the EOL

Participants strongly supported the scientific rationale for combination approaches in HIV cure research at the EOL, highlighting their potential to enhance effectiveness and reduce the risk of viral escape. While recognizing that these studies are not designed to offer direct clinical benefit to PWH at the EOL, many viewed participation as a meaningful contribution to advancing science. The concept of synergy, i.e. multiple strategies working together to achieve more than each could alone, was particularly compelling. Some participants pointed to prior success with combination ART as a model, suggesting that cure research should follow a similar path.

*Right from the early days of combination therapy is that the combination of approaches are very likely to be the most effective, since it avoids all the mutation problems or minimizes the problem of viral escape. So, yes. Combination approaches should definitely be one of the ones that are considered.* – Cisgender Man, White.

*Yeah, that’s synergy, right? How things work together to be more than, you know, what they are separately. And that may be that’s where the treasure would lie in a synergistic approach.* – Cisgender Female, White.

Some perceived that the EOL context was well-suited for testing these approaches, particularly if risks were monitored and the research design was clear.

*I think the kitchen sink is needed. And as you get to the end of life, you know, we need to kitchen sink it. And I don’t think that people have that much of a reticence to engage in that. Because you know, it’s the end of your life. It’s all been a gift anyway, you had no life, you know, since 1995, it’s like, whoa, we’ve outlived our ticket.* – Cisgender Female, Mixed Race.

Others voiced hesitation, citing the added complexity of multiple interventions, concerns about quality of life, and the limited time remaining to observe meaningful effects. For some, the idea of joining a more intensive protocol appeared burdensome, especially after years of managing other health issues.

*It sounds too confusing, just to all of a sudden you got two things going on instead of just one. Yeah. And I’m sure it’s doable. But once again, the knowledge you have to get, but I would think, initially, I would say to you, that sounds a little confusing. I’m already doing three things. What more do I have to do now? You know, I used to just take two pills in the morning. So I hesitate.* – Cisgender Man, White.

*You know, it might, that medicine might have to take two weeks in my body. But if the person that’s end stage doesn’t have three days left, that’s not going to give me the accurate data that I’m looking for.* – Cisgender Female, Black.

While some questioned the practicality of combination approaches at EOL, especially in advanced illness, others welcomed the opportunity to advance HIV cure science. Acceptability was shaped by perceptions of scientific value, personal capacity, and the clarity of the research design.

### Ethical and practical considerations in interventional HIV cure research at the EOL

Generally, participants supported the opportunity to contribute to scientific progress, they also acknowledged the practical, physical, and ethical factors that must be addressed to ensure equitable and meaningful participation.

### Diverse perspectives on ATIs during interventional trials at EOL

In the context of interventional HIV cure research at the EOL, many participants viewed ATIs as acceptable when framed as a contribution to scientific progress, rather than for personal clinical benefit. Participants understood that voluntarily stopping ART would help researchers better evaluate the impact of experimental interventions on viral rebound and reservoir activity. For some, the limited time left to live made participation in such studies and the risks involved reasonable, provided that the decision was voluntary and supported by clear communication and ethical oversight.

Several participants emphasized that PWH nearing the EOL should have the autonomy to discontinue ART if it no longer aligned with their personal goals for comfort or quality of life. Comparing this to decisions to stop chemotherapy or other treatments, they stressed that such choices should reflect individual values and priorities, especially at the EOL.

*If they know that they’re going to die, why continue [ART] to take it? There is no need to keep taking it and [this] should be independent to take decision to do ATIs or not.* – Cisgender Female, Black.

For others, ATIs were not only acceptable at the EOL but desirable under certain conditions. Some perceived ATIs as a means to ease the burden of lifelong adherence, reduce medication fatigue, or symbolically “let go” at the EOL. They noted that the risk of viral rebound was unlikely to result in significant discomfort or clinical deterioration within the limited time remaining.

*If an end-of-life study participant is defined as somebody who is probably within six months of end of life, then really the risks of interrupting HIV treatment are really not very great, because I mean, even if there is a, you know, a major rebound of a virus, the risks are lower at the end of life.* – Cisgender Man, White.

However, not all participants agreed. One respondent strongly opposed stopping ART near the EOL, citing fears of accelerated decline, worsening health, and the loss of the stability that ART had provided for decades. This view reflected a deep attachment to treatment and a desire to maintain as much physical control and well-being as possible, even in the final months of life.

*Oh, no, they should never stop, never stop, you need to know. You’re on them for a reason. You know, you’re stopping and you’re deteriorating in your own body?* – Cisgender Female, White.

Overall, participants held diverse views on ATIs at the EOL, with acceptability shaped by personal health, values, and a desire to contribute to HIV cure science, balanced against concerns about comfort, control, and the meaning of stopping ART.

### Safeguards for ATIs in interventional research at the EOL

Participants who supported ATIs emphasized the need for safeguards to minimize risks. They stressed that any decision to stop ART should be clinically supervised, closely monitored, and, when possible, reversible to prevent unnecessary health deterioration. These measures were considered essential for maintaining trust and ensuring participant safety, even at the final stage of life.

*What are my safeguards? Again, that’s something that you got to determine with each thing. As a non-scientist, I mean, I look at, for me, you know, how low are my T cells? How low is, you know, I mean, as they start getting down under 200, you know, it’s getting a little dicey there.* – Cisgender Man, White.

*There should be close monitoring and reversibility. So if the HIV is reactivated and begins affecting the T cells or other immune functions, there must be a way to reverse that.* – Cisgender Male, Asian.

### Physical and logistical challenges

Participants stated that an individual’s physical condition would determine their ability to engage in HIV cure research at the EOL, with some procedures, such as biopsies or apheresis, being too burdensome for those in declining health. Concerns were also raised about pain and discomfort, with some questioning whether undergoing additional procedures was worthwhile in their final days.

*To do an apheresis or gut biopsy lymph donation that necessarily requires at least probably one day of inpatient. So that might just not be feasible at the end of life.* – Cisgender Man, White.

### Risk tolerance and participation in interventional trials

Some participants expressed a willingness to participate in high-risk experimental interventions, viewing them as an opportunity to contribute to scientific progress or as a final option for potential benefit. One participant emphasized that PWH at EOL may have little to lose, making them more receptive to interventional research.

*You gotta have human trials. Right. And, and yeah, those of us who have reached that point where, you know, we’re beyond hope and we’re gonna die anyway.*– Cisgender Male, White.

Despite this willingness, this participant acknowledged the deeply personal nature of such decisions, noting that the experience of discomfort significantly shapes perspectives on participation.

*If you can make a body clear the HIV with a gene therapy, that doesn’t cause other problems… that to me sounds like something that would not increase suffering at the end of life. So… yeah, let’s try that. Yeah, let’s do that… Or, you know, I’m just done. I’m just worn out. I’m tired of the battle – Cisgender Male, White*

*And it’s, again, it’s very individual.* – Cisgender Male, White.

This perspective highlights both the willingness to take risks in the pursuit of scientific knowledge and the importance of ethical considerations in involving PWH in interventional research at EOL.

## Discussion

Our study uncovered the complex perspectives of older PWH on hypothetical interventional HIV cure research at the EOL, revealing a nuanced interplay of altruism and ethical considerations such as autonomy, informed consent, perceived risk, and the balance between scientific value and personal well-being. Participants expressed a willingness to contribute to interventional HIV cure studies at EOL, including those involving ATIs, driven by a strong desire to advance science and benefit future generations. However, concerns about personal risks, logistical burdens, and the uncertainty of outcomes tempered their enthusiasm. Participants expressed varied views on specific HIV cure-related strategies at EOL. There was overall skepticism surrounding LRAs, and enthusiasm for block-and-lock strategies, which were perceived as promising due to their potential to replicate the durable viral control observed in exceptional elite controllers [49]. Immune-based and gene therapies were met with cautious optimism, while combination approaches were regarded with particular interest for their potential to maximize efficacy through synergistic effects. These findings emphasize the need for ethical, patient-centered cure studies that minimize harm while advancing the scientific agenda, ensuring that LTS voices shape future interventional HIV cure research at the EOL.

Participation in hypothetical interventional HIV cure research at the EOL was often motivated by altruism and a desire to advance science, despite the lack of anticipated personal clinical benefit [38, 39, 50, 51]. This motivation was intertwined with LTS’ recognition of the scientific value of their bodies in advancing knowledge on HIV persistence and HIV cure strategies. Though aware they would not derive direct clinical benefit from research, participants found emotional and psychological fulfillment in knowing their contribution might help future generations, reinforcing findings from previous similar observational research [11, 15, 23, 24]. 

Beyond altruism, autonomy emerged as a critical concern, as participants were aware of historical exclusion from trials due to their age or illness [25]. Older individuals with comorbidities and those nearing the end of life are frequently excluded from HIV cure trials due to concerns about generalizability of results and fears that adverse events in these populations could complicate future regulatory approvals, including those by the U.S. Food and Drug Administration (FDA) [2]. Being given the opportunity to participate in EOL research was perceived as an affirmation of their agency, challenging traditional ethical concerns that categorize terminally ill individuals as inherently vulnerable [16, 22]. Participants’ trust in researchers to utilize their contributions ethically underscores the importance of adhering to rigorous ethical guidelines, ensuring their autonomy is respected while advancing scientific progress [52, 53]. 

Participants’ perspectives on future HIV cure strategies at the EOL highlight the potential of this research to inform broader cure efforts, with the greatest acceptability observed for approaches perceived as lower risk and supportive of overall health. Participants’ general endorsement of testing LRAs at EOL reflects a pragmatic understanding of the need to disrupt viral latency, a major barrier to a cure. While prior LRA trials have shown limited success in reducing the viral reservoir [30], ongoing research suggests that pairing LRAs with immune-based therapies could enhance their efficacy [32, 54]. Participants recognized that reactivating latent virus alone is insufficient without a mechanism to eliminate it. Their willingness to undergo LRA-based interventions, despite no personal benefit, underscores both their scientific curiosity and their altruistic commitment to advancing this approach. In contrast, block-and-lock strategies were widely embraced by LTS due to their potential to suppress HIV indefinitely without requiring elimination. This approach, which seeks to keep the virus dormant in the long-term, appealed to participants as a promising strategy similar to the natural control seen in exceptional elite controllers [30, 31, 49]. Though still in its infancy, block-and-lock strategies could provide a viable alternative for aging or those unable to tolerate more invasive interventions [31]. Similarly, participants viewed immune-based strategies, such as bNAbs and therapeutic vaccines, as essential components of a cure regimen. Recent studies showed that certain bNAb combinations can sustain viral suppression off ART, supporting the idea that immunotherapies could help control HIV in the absence of medication [55–57]. However, our participants raised concerns about immune function in aging PWH, highlighting the need for further research on the effectiveness of these strategies in older populations. Their nuanced understanding of immunological limitations, combined with enthusiasm for these approaches, underscores the importance of developing cure strategies that account for age-related immune decline. Cell and gene-based therapies prompted a mix of optimism and caution, reflecting broader debates in the field. While some participants viewed gene editing as a revolutionary tool for eliminating HIV, others were hesitant about its risks and ethical implications [30, 58]. Emerging gene-editing techniques, such as CRISPR, show promise for modifying host cells to resist HIV, though these approaches remain in early experimental stages often being tested in otherwise healthy volunteers [34]. Participants’ concerns about feasibility and safety align with scientific discussions about off-target effects and long-term risks, reinforcing the need for rigorous oversight in gene therapy trials. Across all modalities, participants emphasized that combination approaches are likely necessary for a cure, echoing expert consensus that no single intervention is sufficient [52]. The willingness of LTS to undergo multi-pronged strategies presents a unique opportunity for researchers to test novel combinations in ways that are not always possible in conventional trials. Based on their views, domains of cure research perceived as most suitable for LTS participation at the EOL included lower-risk, less invasive strategies such as block-and-lock approaches and immune-based therapies (e.g., bNAbs). Participants were more cautious about intensive or unproven interventions like gene editing and complex combination regimens. Additionally, ex vivo testing, where biological samples are studied outside the body, may offer a promising alternative for those concerned about physical burden, and should be further explored.

ATIs are central to HIV cure trials, but their feasibility and ethical implications at the EOL remain complex [59]. In our study, most LTS were willing to stop ART at the EOL as part of hypothetical interventional trials, prioritizing scientific contribution over concerns about viral rebound or immune decline. This perspective contrasts with that of younger healthier PWH who often find ART interruption a challenge, mainly due to the fear of transmitting HIV to sex partners [53]. This finding also aligns with prior research indicating that individuals with advanced illness tend to accept greater research-related risks [53, 60]. However, our findings emphasize that interventions should be safe and not cause discomfort or significant symptoms before testing them in PWH at the EOL and there is need for transparency regarding symptom management and opportunistic infections, as some participants expressed concerns about potential discomfort in their final days [61]. In addition to robust safeguards and clinical monitoring, protocols should include plans for managing adverse events such as cytokine release syndrome or other intervention-related complications to ensure participant well-being. Additionally, conducting interventional HIV cure research at the EOL would require overcoming logistical challenges, particularly in ensuring that experimental interventions are administered effectively within the constraints of terminal illness. Unlike observational studies, interventional trials demand real-time monitoring and clinical assessments, necessitating close collaboration between research teams, healthcare providers, and hospice organizations [62]. Despite these complexities, our study found that participants remained deeply committed to advancing HIV cure research at the EOL, underscoring the need for flexible, participant-centered study designs. Strategies such as home-based visits, telemedicine consultations, and mobile phlebotomy services can mitigate logistical barriers while maintaining scientific rigor [63, 64]. Successful models, like the UCSD Last Gift program, demonstrate that with strategic planning and robust community engagement, high-quality observational research can be conducted at the EOL [24]. Lessons learned from these models can lay the foundations to establish interventional cure research program at the EOL. Future studies should integrate adaptive methodologies to ensure that participants’ final contributions are both ethically sound and scientifically valuable.

Conducting interventional HIV cure research at EOL necessitates proactive and careful ethical considerations to protect participants’ rights and well-being. Ensuring robust informed consent is paramount, requiring participants to fully understand the research purpose, procedures, potential risks, and potential benefits [25]. This process should be dynamic, accommodating the evolving health status and preferences of individuals nearing the EOL. Moreover, involving the next-of-kin (NOK) or loved ones in the research process should occur only with the explicit approval of the PWH [11], respecting their autonomy and privacy. Such involvement can provide additional support but must not override the participant’s wishes. Implementing these ethical safeguards helps prevent exploitation and ensures that the altruistic contributions of PWH at the EOL meaningfully advance scientific knowledge while honoring their dignity and personal choices.

Table 3 summarizes recommendations suggested by diverse LTS on intervention HIV cure research at the EOL across the U.S.

**Table 3:** Summary of recommendations suggested by LTS on interventional Hiv cure research at the EOL

Key Considerations	Refined Actionable Strategies
**Ethical and Informed Consent Protocols**	• Develop informed consent frameworks ensuring comprehension of risks, expected outcomes, and participant rights. • Engage bioethicists, regulators, advocates, community advisors, and next-of-kin/loved ones to co-design accessible informed consent and decision-making support materials. • Implement reassessment mechanisms for ongoing informed decision-making as participants’ health evolves. • Provide educational resources tailored for older adults about EOL research.
**Guidelines for Analytical ART Interruption in EOL Research**	• Address psychological and clinical concerns through tailored participant counseling before analytical ART interruption. • Evaluate immune responses and viral rebound dynamics in aging populations to guide EOL-specific ART policies. • Develop structured protocols for medical oversight, symptom management, and voluntary ART reinitiation. • Conduct sub-studies on physiological impacts of viral rebound in aging populations.
**Adapting Study Designs for Feasibility**	• Ensure flexibility in research participation, offering home-based, hospice-supported, and telehealth-integrated options. • Reduce participant burden through streamlined procedures with minimal disruption to palliative care. • Establish contingency plans for participant deterioration, ensuring dignity and data integrity.
**Managing Risks of Latency-Reversing Agents (LRAs)**	• Limit LRAs to low-toxicity candidates with manageable side effects in older populations. • Conduct preliminary safety testing in younger populations before extending trials to older participants. • Implement real-time safety monitoring and immediate ART reinitiation pathways if adverse effects arise. • Conduct post-mortem assessments to evaluate LRAs’ effects on latent reservoirs.
**Enhancing Safety of Immune-Based Approaches**	• Prioritize immune-based therapies with lower risk of overactivation, tailored to aging immune systems. • Evaluate the feasibility of bNAbs and therapeutic vaccines specifically in LTS and EOL cohorts. • Investigate immune-based strategies’ potential synergy with latency-reversing agents in EOL settings.
**Ethical Oversight in Gene Therapy Interventions**	• Ensure rigorous safety validation of gene-editing interventions before EOL implementation, with clear communication about risks and unknowns. • Address participant concerns about potential unintended consequences by providing transparent data on prior safety trials and ethical oversight. • Limit gene therapy trials at EOL to interventions with established safety profiles to minimize uncertainty and potential harm.
**Feasibility and Implementation of Block-and-Lock Strategies**	• Validate long-term effectiveness and safety of block-and-lock approaches in preclinical models. • Determine feasibility for individuals with short life expectancy by assessing stability of viral suppression. • Communicate realistic expectations, addressing whether ART may still be required long-term.
**Designing Effective Combination Cure Strategies**	• Identify optimal intervention pairings to balance efficacy and participant burden. • Ensure robust safety and feasibility trials before introducing multi-modal interventions at EOL. • Establish systematic data collection frameworks to maximize the scientific impact of EOL combination studies.
**Expanding Awareness and Accessibility of EOL Cure Research**	• Leverage HIV advocacy networks, hospice organizations, and patient-led initiatives for outreach. • Deploy peer navigators and tailored educational resources. • Address misconceptions through ongoing community dialogues and research transparency efforts.
**Policy**,** Structural**,** and Regulatory Support**	• Advocate for policy adjustments supporting ethical EOL interventional research. • Secure long-term funding for EOL cure trials to ensure sustainability and scientific rigor. • Promote regulatory adjustments to accommodate accelerated approvals for promising interventions.

### Limitations

This qualitative interview study has several limitations. First, we interviewed a subset of PWH who were willing to discuss participation in HIV cure research at the EOL, which may introduce selection bias. Although we aimed to recruit a diverse national sample across racial and ethnic groups, Hispanic individuals were notably absent, reflecting the broader underrepresentation of Spanish-speaking populations in HIV and cure research. Additionally, as the study was conducted in the U.S., the findings may not be generalizable to other settings. The EOL PWH population is also heterogeneous; future studies should explore perspectives across varied clinical, social, and cultural contexts. Another limitation is that we focused our inquiry on hypothetical in vivo experimentation. It is likely that ex-vivo studies, using cells donated at the EOL and testing interventions outside the body, would be more acceptable to participants, and should be explored in future research. We also did not collect systematic data on participants’ prior involvement in interventional research, which may have influenced their openness to risk. Individuals familiar with research may be more willing to participate in complex studies, whereas those with limited experience could hold more cautious views. Finally, participants’ responses reflect hypothetical willingness to participate in EOL HIV cure research. Since the interventional research paradigm remains theoretical, perspectives may shift once such studies are implemented. As this field develops, continued engagement with key stakeholders such as regulatory authorities and IRBs will be critical to ensure appropriate ethical safeguards. This underscores the importance of a collaborative ethics model, such as the one proposed by Lunshof et al., to guide responsible and inclusive research design [65]. 

## Conclusions

This study highlights the broad willingness of diverse LTS to participate in EOL HIV cure research, motivated by altruism and a strong commitment to scientific progress. Participants opposed excluding older PWH from cure research at the EOL, underscoring the need for inclusive study designs that acknowledge their unique contributions. While many LTS supported interventional studies, including ATIs, they emphasized the need for robust ethical safeguards, informed consent, and measures to prevent undue discomfort at EOL. Perspectives on cure strategies varied: LRAs raised concerns about safety, block-and-lock strategies were viewed as promising, immune-based therapies and gene-editing approaches were met with cautious optimism, and combination strategies were favored for their potential efficacy. As HIV cure research advances, ensuring the meaningful engagement of LTS in EOL studies is essential to uphold ethical standards and maximize scientific impact.

## Electronic supplementary material

Below is the link to the electronic supplementary material.

Supplementary Material 1

## Data Availability

All the data is presented within the manuscript and in supplementary file. If you need more information, please feel free to reach out corresponding author at kdube@health.ucsd.edu.

## Abbreviations

ART: Antiretroviral Treatment/Therapy
ATI: Analytical Treatment Interruption
bNABs: Broadly Neutralizing Antibodies
CBOs: Community-Based Organizations
CRISPR: Clustered Regularly Interspaced Short Palindromic Repeats
DARE: Delaney AIDS Research Enterprise
EOL: End of Life
FDA: Food and Drug Administration
HARP-PS: HIV + Aging Research Project – Palm Springs
HIPAA: Health Insurance Portability and Accountability Act
IDIs: In-Depth Interviews
IRB: Institutional Review Board
LTS: Long-Term Survivors
LRAs: Latency Reversal Agents
PWH: People with HIV
RID-HIV: Reversing Immune Dysfunction for HIV-1 Eradication
NMAC: National Minority AIDS Council
SFAF: San Francisco AIDS Foundation
UCSD: University of California San Diego
U.S: United States
