# Supplementary material for: Motivations, acceptability and ethical considerations for interventional HIV cure research at the end of life: perspectives from long-term survivors of HIV in the United States
Source: BMC Med Ethics. 2025 Aug 23;26:112. doi: 10.1186/s12910-025-01253-x (PMC12374411; doi:10.1186/s12910-025-01253-x)
Supplement: Supplementary file 1 — Supplementary Material 1 [file 12910_2025_1253_MOESM1_ESM.docx]

**Supplementary Table S1: Additional Quotes – Perceptions of Long-Term Survivors of HIV about Hypothetical Interventional HIV Cure Research at End-of-Life (United States, 2023 – 2024)**

| **Themes** | **Participants** | **Quotations** |
| --- | --- | --- |
| **Motivations and Autonomy in Research Participation in Interventional HIV Cure Research at the EOL** | | |
| **Altruism and Contribution to Science** | Cisgender Female, Mixed Race | *I want to do that [participate in interventional cure research at the EOL], if it can help someone else.* |
|  | Cisgender Man, White | *The interventions should definitely be considered, not just observational studies.* |
|  | Cisgender Female, White | *Well, sure, I’d be willing to try anything. I’ve had it for 23 years, so I'm willing to try anything to help others.* |
|  | Cisgender Female, Mixed Race | *I went through all this so we could learn, you know, like, let’s make a good thing out of it.* |
|  | Cisgender Male, White | *If you could find a way to knock the virus out of someone who’s had it for 40 years, we would all line up for that.* |
| **Scientific Value and Willingness to Participate** | Cisgender Man, White | *I think they [these studies] could be really important, especially if they were dying… it will be very valuable to have markers attached to lymphocytes, reinjected, and then establish after death, to what extent they had reached all the various tissue types in the body.* |
|  | Cisgender Male, White | *Why exclude a certain group of people from something that can help millions?* |
|  | Cisgender Man, White | *To be said, you can't participate because you're at the end of life, or you are sick, that devalues me as a person. And I resent that.* |
| **Autonomy and Informed Choice** | Cisgender Female, White | *They should be given a choice, yes, or no? Are they willing to do it? If they're not, then there is nothing you can do.* |
|  | Cisgender Female, White | *Everyone should have the right to make these decisions. They should be offered, and then they can decide what’s right for them.* |
|  | Cisgender Man, White | *A professional, talking to your medical personnel. Definitely a big thing with this, if you’re going to try and talk to someone about things that they are going through, talk to someone who’s done it.* |
|  | Cisgender Male, White | *I think similar kinds of interactions show people what’s possible... what we hope to gain, share the goals. People know the goals; they need to be more willing to buck up and be there.* |
|  | Cisgender Male, White | *Making sure that people are making informed decisions, making sure that they are their own decisions, they're not a caretaker's decisions.* |
|  | Cisgender Male, White | *You might convince me to try gene therapy if it’s not going to hurt me.* |
| **Acceptability of Testing Specific HIV Cure Research Interventions at the EOL** | | |
| **Perceptions on Testing LRAs at the EOL** | Cisgender Male, Asian | *Yes, definitely. Yeah. Because that reawakening of HIV and then killing them with the already existing drugs is the solution.* |
|  | Cisgender Male, Black | *Will this shorten my life? That would be my main concern. Once you awaken the beast, can you put them back to sleep?* |
|  | Cisgender Male, Black | *They always warn you about not keeping to your protocol… and they always say if you go off your protocol, you're going to become very resistant to medications. And if you've had real concerns about or had opportunistic infections, it may create more, right? Or new ones, you know, so, and with the age factor, bodies are more vulnerable to that sort of thing. So that's gonna be a hard sell.* |
|  | Cisgender Female, Black | *If you’re end stage and you’re dying, I would think that your body is slowly shutting down, so how much of the reservoir would wake up? I don’t know. It couldn't do any more damage than what the virus has already done to your body at this stage in life.* |
|  | Cisgender Man, White | *I have no problem with a latency-reversing drug... I mean, okay, I’ve got my, I’m happy. If every three months, I’m under 20 on my viral load test, but that doesn’t really give me the big picture... I don’t have a problem with that.* |
| **Perceptions about Block and lock Approaches at the EOL** | Cisgender Female, Black | *Most definitely. I think that will be I mean, just the interest that you know that sparked in me to know rather not, you know, if it [virus] can be contained, whatever I think will just be a great research and study. Yes.* |
|  | Cisgender Male, Black | *I think it'd be great if you if you could keep it dormant for the rest of my life. You know? Yeah. Especially if I were 20 years younger.* |
| **Perceptions about Immune-Based Strategies at the EOL** | Cisgender Man, White | *That I’d go for. I'm a big fan of the immune system... We've been loading our gut with HIV meds all these years. What microbiome do I have that's different than someone else? You know, maybe it's something that's going to give something else? So I think it would be great now.* |
|  | Cisgender Male, White | *Definitely. I mean, you know, that's the decline of the immune system. The immune system ultimately probably kills all of us, right? Why not study things that are going to boost that?* |
|  | Cisgender Female, Mixed Race | *Strengthening the immune system is a new science. And we need more of it. But strengthening the immune system, would that mean that would be life extension? Perhaps?* |
| **Perceptions about cell and gene Approaches at the EOL** | Cisgender Female, White | *I think that sounds interesting as well, I would try. I would try that [gene therapy]. I mean, yeah, I guess once it's already in there, though, it's in there. But I imagine there's a way that researchers can determine that there's no new virus getting in, I suppose.* |
|  | Cisgender Male, White | *The [regulatory bodies] has got to be prepared to accept probably some higher adverse event counts than, you know, percentages than usual. Obviously, patients have to be, you know, accepting of the possibilities. Many of us that are, we've been sitting around all these years, and surviving all these years, and where, you know, we're getting closer and closer to the end. And, you know, some of us would really welcome these opportunities, even with the risks that [gene therapy] presented.* |
| **Perceptions about Combination Approaches at the EOL** | Cisgender Male, White | *I mean, seriously, the advances that have been made in the last 15 years in gene research and CRISPR. And, you know, all these things. I mean, it is sort of, it is sort of trippy. But yeah, so why not combine it all, or, you know, or different parts of it all? And see if it may come to help come to a place where there is a cure.* |
|  | Cisgender Male, White | *If researchers have a strategy to figure out which, when, they're obviously doing multiple approaches, if they're able to winnow out the results to get good data, that's useful. If they have a plan for that. And then why not?* |
|  | Cisgender Male, Black | *For me where I am in my life right now, I don't know if I want to go through another [combination] protocol. I'm more concerned about the fact that I want to keep mobility as long as I can. And I've got back issues with degenerative disc disease and that's going to keep me challenging my mobility. So, if I go on another protocol, will that affect my quality of life? Or will it improve my quality of life?* |
|  | Cisgender Male, Asian | *Yeah, there'll be more reluctant to do mixture of things.* |
| **Ethical and Practical Considerations in Interventional HIV Cure Research at the EOL** | | |
| **Diverse Perspectives on ATIs during Interventional Trials at EOL** | Cisgender Female, White | *Well, to me, that's a personal choice. And if it to me, it seems the same as people who have decided that they've tried everything they want to do, and they don't want to be like on chemo anymore. They just want death to come naturally, or whatever their situation might be. And they've just decided that they're done fighting, and they're ready to go. And I think people should be allowed to do that. That should be their choice. So yeah.* |
|  | Cisgender Female, Black | *I think it's left up to the individual. Because, you know, after a point in time, you do stuff so much out of habit that it's just like it's a natural process that you have to continue to do, but you feel like you left forgot to do something. So I think if they want to stop, yes, but if they don't want to stop, and if they feel comfortable enough and strong enough that that's what they can still swallow with no problem, then your main idea is to make them comfortable. It's about them. It's no longer about what you want for them. It's about what they want for themselves.* |
|  | Cisgender Man, White | *Yeah, sure. I mean, I’ve thought a lot about this end-of-life stuff. So I don’t, you know, I think if I ended up in hospice or near there, I’d probably stop taking my meds.* |
|  | Cisgender Man, White | *Sometimes I think to myself, you know, what would it be like to spend the last six months of my life without taking ART, without that constant? Because, for me, I'm still on pills... So, in some way, it might be kind of like an additional relief at the end of life, to not be on it.* |
|  | Cisgender Female, Asian | *How much time do they have? Because you must know how much time do they have? If they're like, okay, if I have like, six months, too long. And do I want to have that done for me? I don't know. Because what about the issues? I've been having my virus get overloaded or something. It's no big deal, because I'm going to die anyway.* |
| **Safeguards for ATIs in Interventional Research at the EOL** | Cisgender Man, White | *How high my viral load can go, how long you can stay that high, how low my T cell count can go. And then, I guess, you know, it would be the appearance of any other opportunistic infection.* |
|  | Cisgender Female, Mixed Race | *Oh, we should have good supervision. You know, for them good supervision that they can, you know, check in if they feel something’s gone awry.* |
| **Physical and Logistical Challenges** | Cisgender Man, White | *It’s going to depend very much on people’s, you know, physical condition, I would say.* |
|  | Cisgender Male, White | *How much pain is it going to cause me? You know, I know I’m at the end here, I know that this is not going to save me.* |

EOL: End of Life
